# A fatal SARS-coronavirus-2 induced bone marrow aplasia complicated with invasive fungal infection and severe neutropenic enterocolitis

**DOI:** 10.1186/s12879-022-07599-6

**Published:** 2022-08-09

**Authors:** Ali Amanati, Seyyed Bozorgmehr Hedayati, Mazyar Ziyaeyan, Alireza Honar, Reyhaneh Dashtianeh, Negin Rabiei, Nasrin Saki, Leila Karami

**Affiliations:** 1 Professor Alborzi Clinical Microbiology Research Center, Shiraz University of Medical Sciences, Shiraz, Iran; 2 The Hematology Research Center, Shiraz University of Medical Sciences, Shiraz, Iran; 3 Shiraz University of Medical Sciences, Shiraz, Iran; 4 Dermatology Department, Shiraz University of Medical Sciences, Shiraz, Iran; 5 Molecular Dermatology Research Center, Shiraz University of Medical Sciences, Shiraz, Iran

**Keywords:** SARS-CoV-2 infection, Pancytopenia, Bone marrow-induced aplasia, Aplastic anemia, Prognosis

## Abstract

**Background:**

Immunization against the coronavirus disease 2019 (COVID-19) began in January 2021 in Iran; nonetheless, due to a lack of vaccination among children under 12, this age group is still at risk of SARS-CoV-2 infection and its complications.

**Case presentation:**

SARS-CoV-2 infection was diagnosed in a 6-year-old girl who had previously been healthy but had developed a fever and pancytopenia. The bone marrow aspiration/biopsy demonstrated just hypocellular marrow without signs of leukemia. She was worked up for primary and secondary causes of pancytopenia. Except for a repeated reactive HIV antibody/Ag P24 assay, all test results were inconclusive. After a thorough diagnostic investigation, the cross-reactivity of the HIV antibody/Ag P24 test with SARS-CoV-2 antibodies was confirmed. The patient did not develop any COVID-19-related signs and symptoms, but she did get a severe invasive fungal infection and neutropenic enterocolitis. She died as a result of disseminated intravascular coagulopathy.

**Conclusion:**

It is critical to recognize children infected with SARS-CoV-2 who exhibit atypical clinical manifestations of COVID-19, such as persistent pancytopenia. SARS-CoV-2 infection can cause severe and deadly consequences in children; thus, pediatricians should be aware of COVID-19’s unusual signs and symptoms mimicking other conditions such as aplastic anemia.

## Background

Severe acute respiratory syndrome coronavirus 2 (SARS-CoV-2) is a coronavirus genus in the family *Coronaviridae* and order *Nidovirales* that causes severe respiratory syndrome [1]. Children can be affected by SARS-CoV-2 infection like all other age groups [2]; however, they are less likely to be symptomatic or develop severe symptoms [3].

While upper and lower respiratory symptoms are common SARS-CoV-2 symptoms, the virus can affect different body sites, and extrapulmonary manifestations, such as gastrointestinal, nervous, cardiovascular, and thromboembolic events, have been reported in COVID-19 patients [4, 5].

Lymphocytopenia has been documented in most individuals with a severe COVID-19 phenotype [6], but SARS-CoV-2-induced pancytopenia is less well understood [7]. Managing patients with SARS-CoV-2-induced pancytopenia can be complex, and clinical and paraclinical findings can be vague, mimicking other diseases such as aplastic anemia, especially in young children [8].

## Case presentation

A 6-year-old girl was admitted to our center, Amir Oncology hospital, a tertiary teaching hospital in Shiraz, Iran, on October 2, 2021, due to pancytopenia. On admission, she was febrile with stable vital signs (heart rate of 80 beats per minute and respiratory rate of 22 beats per minute). Except for some inexplicable bruising on her limbs and a solitary 5 × 5 mm erosion on her lower lip, no specific symptom was found. Table 1 summarizes the lab results throughout the patient’s hospital stay. A trephine bone marrow biopsy demonstrated hypocellularity with three lineage aplasia without signs of leukemia, myelodysplastic syndrome (MDS)/myeloproliferative neoplasm (MPN), or megaloblastic changes. Only 18% of benign immature B cells (hematogenous)-positive for CD10, CD19, CD20, and negative for CD33, CD117, and TdT- were found in bone marrow flow cytometry without any evidence of lymphoma or leukemia. There was no evidence of paroxysmal nocturnal hemoglobinuria (PNH) in peripheral blood flow cytometry phenotyping. The chromosome breakage analysis was used to rule out Fanconi anemia. Dyskeratosis congenita and other rare cause of primary AA was investigated by Next-Generation Sequencing (NGS). It is important to note that our patient was previously healthy and does not exhibit any of the classic signs and symptoms of Fanconi anemia (mostly birth defects, such as abnormal thumbs, skin pigmentation, small heads, small eyes, and cardiac and skeletal anomalies) or dyskeratosis congenita (abnormal skin, nail dystrophy, and oral leukoplakia).

**Table 1:** The patient’s laboratory test results throughout her hospital stay

	Day of admission											
	0	1	4	5	7	10	2	14	17	21	24	25
WBC count (per mm^3^)	1000	580	310	410	330	390	370	270	2040	190	180	200
Hemoglobin (gr/dl)	8.7	7.7	6.7	11.2	10.5	9.6	9.1	7.6	8.3	7.5	9	8.4
Platelet count (× 1000 per mm^3^)	7	26	40	34	8	130	60	8	16	14	14	6
Blood urea nitrogen (mg/dL)	26	12	7		6	6		8	7	8	6	
Serum creatinine (mg/dL)	0.74	0.5	0.6		0.6	0.4		0.4	0.3	0.43	0.4	
Serum sodium (Na) mEq/L	132	135	139		132	132		133	134	134	135	143
Serum potassium (K) mEq/L	4.1	4.1	3.4		4.1	4.6		3.7	4.2	4	3.1	3.8
Serum calcium (Ca) mg/dL		9.4	9		9.5	10		9.6	9.4	9.2	8.7	
Serum Magnesium (Mg) mEq/L		1.75	1.65		1.82	2.19		2.09	2.06	2.02	1.82	
Uric acid μmol/L		2	1.5		2.4	2.1		3	2.5	3	2.3	
Fasting Blood Sugar (FBS) mg/dL	104	97	137		115	116		159	137	151	162	
ESR (mm/hours)		103										
CRP (mg/dL)		3	12						79	82	85	
Alanine aminotransferase (U/L)		55	46		23	19		12	15	25	26	
Aspartate aminotransferase (U/L)		39	42		35	24		13	11	19	13	
Total bilirubin (mg/dL)		0.64	0.46		0.63	0.76		0.34	0.62	0.68	0.81	
Direct bilirubin (mg/dL)		0.26	0.22		0.25	0.26		0.17	0.27	0.34	0.38	
Alkaline phosphatase (IU/L)		585	461		483	401		404	379	331	284	
Serum albumin (mg/dL)		4.5	4.1		4.4	4.7		4.2	4	3.8	3.6	
Lactate Dehydrogenase (LDH) (IU/L)		424	372		406	333		229	238	244	180	

*WBC* white blood cell, *ESR* erythrocyte sedimentation rate, *CRP* C-reactive protein

In an abdominopelvic ultrasonographic examination, no hepatosplenomegaly was detected. A day following admission, a real-time polymerase chain reaction (RT-PCR) assay for COVID-19 was reported positive from a nasopharyngeal swab. Following the primary bone marrow (BM) report, we looked for secondary causes of bone marrow aplasia. The secondary causes of BM aplasia were excluded and the patient was transferred to an isolated room in the COVID-19 ward. Serum folate and vitamin B12 levels were normal (8.5 ng/ml and 459 pg/ml, respectively). For virological markers, both serological and molecular assays were used. Anti-parvovirus B19 antibody (IgM) and blood RT-PCR parvovirus B19 both were negative. She was seropositive (IgG positive, IgM negative) for hepatitis A virus (HAV), cytomegalovirus (CMV), and Epstein-Barr virus (EBV). Her anti-HBs antibody titer was above protective values (318.54 mlU), and she had received full primary immunization against hepatitis B virus (HBV). She was seronegative for hepatitis C virus (HCV). The fourth-generation p24 human immunodeficiency virus (HIV) antigen/antibody assay was reactive for HIV I/II.

Parents were asked about any high-risk behavior, and she was evaluated for HIV-related signs and symptoms by daily physical examination. She has never had blood or blood products infusion. Parents were tested using the fourth-generation p24 HIV antigen/antibody assay which both have negative results.

**Fig. 1:**
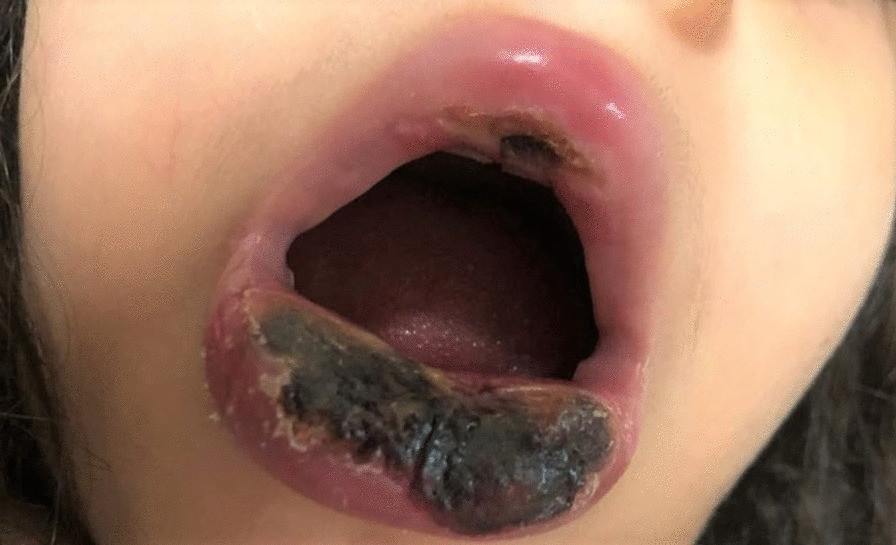
Severe lower lip swelling with erythema and necrosis

**Fig. 2:**
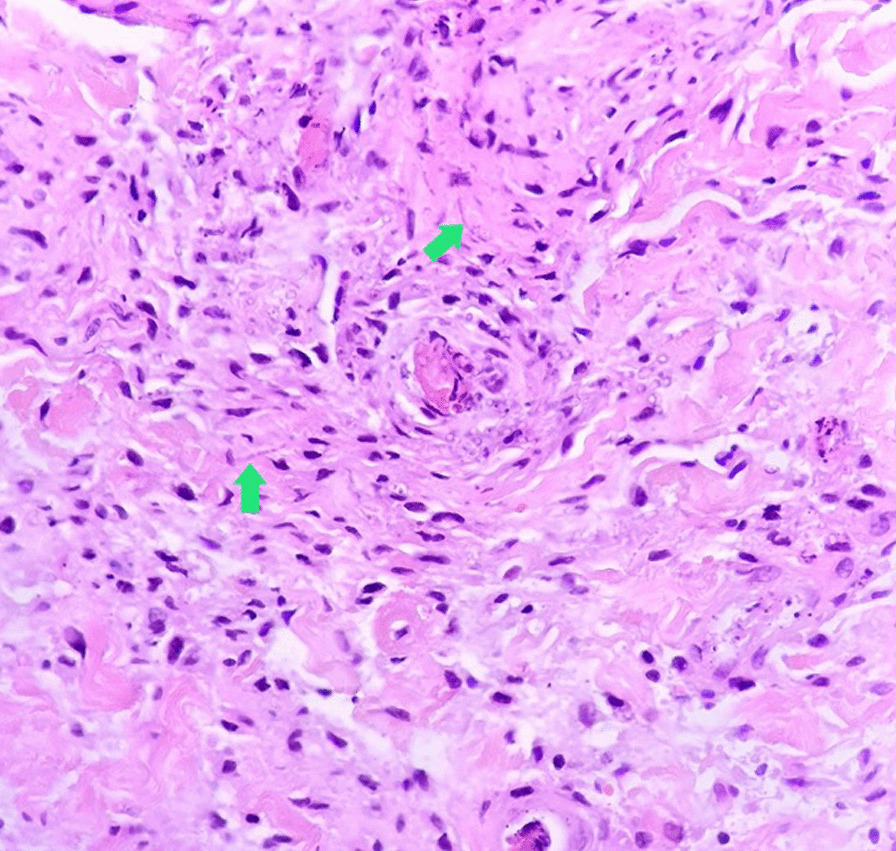
Vascular thrombosis with presence of some short and septate hyphea (arrows) around and in the wall of the vessels (H&E × 400)

The patient was tested for HIV antibody/Ag P24 in Pars virology specialty laboratory in Shiraz, which was reactive again. Several samples were sent to the Shiraz AIDS Research Center on separate days to ensure the test's reactivity which one of them reported as an intermediate result. According to the Iranian national guidance [9], the HIV-1 RNA PCR was tested in the Shiraz HIV research center and the Keyvan Virology Specialty Laboratory in Tehran, both of which were negative. So, the reactive HIV antibody/Ag P24 test was considered a false-positive result due to cross-reactivity with a recent SARS-CoV-2 infection. She was febrile continuously during her admission course. Blood and urine cultures were negative on multiple occasions, and she had monophasic or biphasic high-grade fever bouts despite broad-spectrum antibiotic treatment with piperacillin-tazobactam (100 mg/kg/dose per 6 h) and vancomycin (15 mg/kg/dose per 6 h). Repeated blood sampling revealed a negative serum galactomannan test, and fungal blood culture utilizing BACTEC™ Myco/F Lytic culture vials revealed a negative result. On a spiral computed tomography (CT) scan, the paranasal sinuses, chest, and mediastinum were all unremarkable. Her lip skin lesion had become necrotic, with soft tissue edema dominating (Fig. 1). A dermatologist performed a skin biopsy to rule out invasive fungal infections and vasculitis. On microscopic examination, detached hyperkeratotic layers, acanthosis, submucosal infarction, vascular proliferation, and mild perivascular inflammation were detected. Vascular thrombosis, bleeding, many nuclear bags of dust, fungal short and septate hyphae surrounding and, in the vessels, were also present (Fig. 2).

**Fig. 3:**
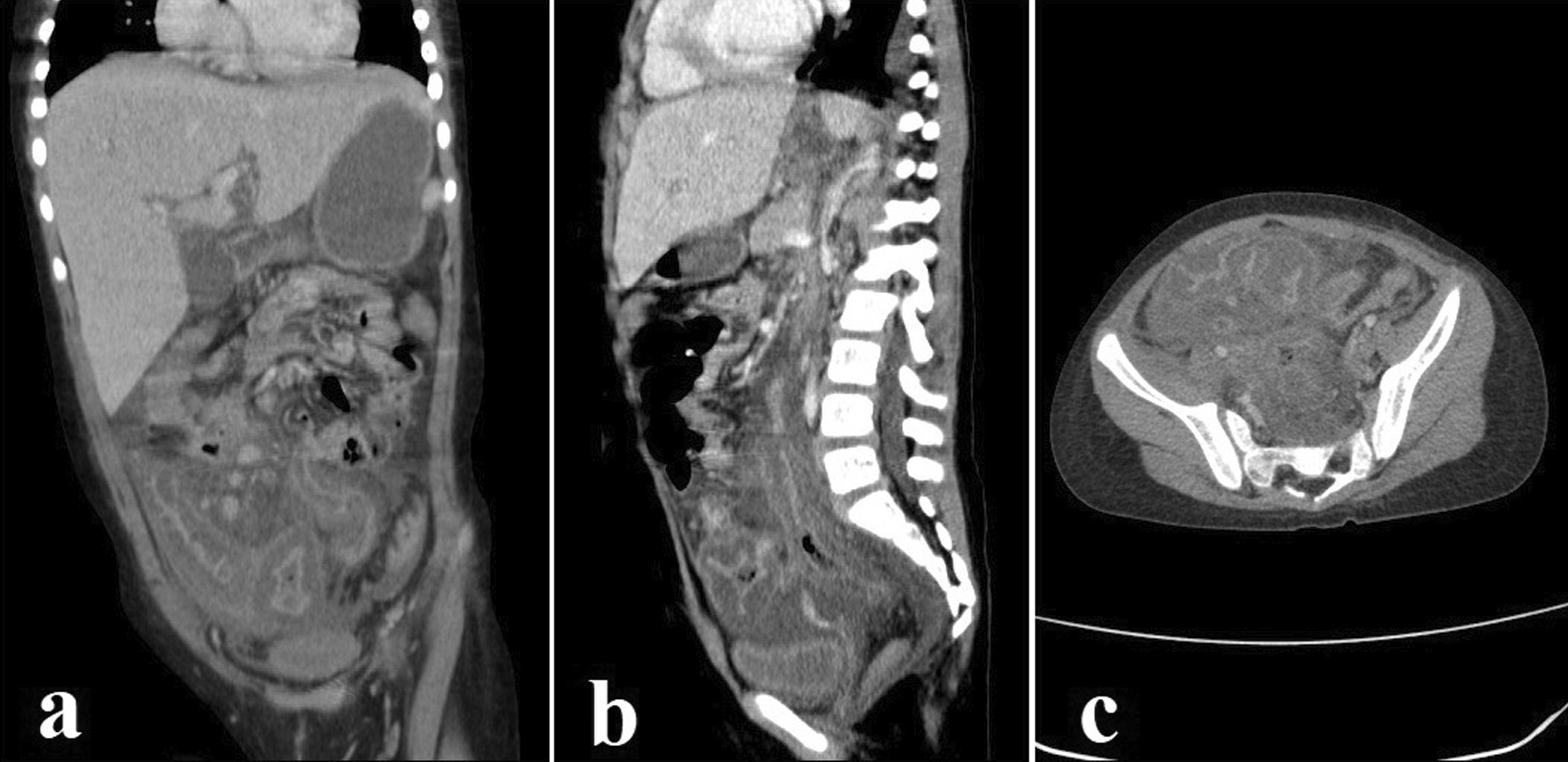
**a**–**c** Coronal, sagittal, and axial reconstruction images through the abdomen and pelvic spiral CT scan after intravenous and oral contrast administration demonstrate mild amount of ascitis in abdominopelvic cavity, significant wall edema of cecum and ascending colon with severe adjacent fat stranding and edema. Also, significant wall edema of sigmoid colon and multiple sub-centimeter mesenteric lymph nodes is seen. Significant haziness of mesenteric fat and peritoneum is seen in favor of peritonitis

Because of the patient's thrombocytopenia (platelet count 30,000/mm^3^), a skin biopsy was obtained and sent for histology. No fungal culture was performed since the tissue sample was very small. Therefore, a broad antifungal agent was preferred to overcome invasive cutaneous *Aspergillosis* and *Mucormycosis*, and liposomal amphotericin B (L-AmB) was started (5 mg/kg/day). After two weeks of antifungal treatment, the patient was still feverish, and the lip lesion had only partially healed. Despite antifungal and antibiotic treatment, the patient experienced new-onset abdominal pain, nausea, and vomiting. The pain was primarily apparent in the right lower quadrant on physical examination, which was due to neutropenic enterocolitis. Her treatment regimen was changed to linezolid, colistin, and voriconazole was added to her antifungal regimen. The results of several abdominopelvic ultrasonographic investigations were inconclusive, and abdominal pain worsened progressively. A surgical consultation was requested, but exploratory laparotomy was postponed due to the patient’s very low platelet count (< 10,000/mm^3^), and the patient underwent an abdominopelvic CT scan with contrast for better evaluation (Fig. 3). After intravenous and oral contrast administration, several axial and coronal reconstruction images through the abdomen and pelvis show mild ascites in the abdominopelvic cavity, severe colitis, multiple sub-centimeter mesenteric lymph nodes, and peritonitis. The patient’s clinical status deteriorated due to the advent of acute abdominal distension and respiratory distress, necessitating admission to the pediatric intensive care unit (PICU). The patient was intubated and put on mechanical ventilation less than 24 h after being admitted to the PICU. On November 1st, 2021, she developed disseminated intravascular coagulation (DIC) and passed away from multi-organ failure (30th day of admission).

## Discussion and conclusions

We describe a clinical case of proven SARS-CoV-2 infection in a 6-year-old girl with persistent pancytopenia and invasive cutaneous fungal infection. The patient was previously healthy and had no family history of congenital disorders in her first-degree relatives. The patient had never been exposed to any toxins and had no prior medical history at the time of admission. COVID-19 infection was confirmed by positive nasopharyngeal PCR. She had a progressive necrotic skin lesion in the lower lip. With suspicion of fungal infection, the patient underwent a skin biopsy of the necrotic lesion. The direct examination revealed fungal elements. The patient was treated with intravenous L-AmB and voriconazole. Despite broad-spectrum antibacterial and antifungal treatment, the patient died of severe neutropenic enterocolitis.

### Potential mechanisms of bone marrow-induced aplasia after SARS-CoV-2 infection

The SARS-CoV-2 infection has different effects on the host's immune responses. Varying degrees of decrease in the absolute T lymphocytes count (CD3 + T, CD4 + T, or CD8 + T cells) are observed in mild to moderate COVID-19 phenotypes, but in more severe cases, the decrease in T lymphocytes count increases significantly [6, 10]. Low CD8 + T and B cell counts and increased CD4/CD8 ratio have been identified as predictors of poor response to treatment. Interferon-gamma (IFN-γ) production by CD4 + T cells has also been lower in severe disease phenotypes [11, 12]. Multi-system inflammatory syndrome (MIS) is one of the other rare SARS-CoV-2 complications in children and adults linked to disturbed innate and adaptive immune responses, characterized by a cytokine storm [13, 14]. Despite the well-known consequences of SARS-CoV-2 infection on the host immune responses, bone marrow (BM) induced aplasia is less known. Many viral infections can impact hematopoiesis by directly influencing the function of hematopoietic stem and progenitor cells (HSPCs) or indirectly by inducing different patterns of cytokines and chemokines. In the acquired aplastic anemia (AA), it has been suggested that oligoclonal CD8 + T cells could affect hematopoietic tissue and BM failure by IFNγ and Tumour necrosis factor α (TNFα) production leading to hematopoietic cell death [15]. IFNγ and TNFα increased during cytokine storms in patients with hemophagocytic lymphohistiocytosis (HLH) [16]. Similarly, a local cytokine storm has been described in the infected lung tissue in severe COVID-19 [17]. So, SARS-CoV-2 induced BM aplasia theoretically could be occurred by impacting the hematopoietic process; however, further studies need to investigate cytokine patterns following SARS-CoV-2 infection for better insight.

### Primary versus secondary aplastic anemia

Few reports describe the clinical features of SARS-CoV-2 infection in previously known AA cases and those with idiopathic AA diagnosed after documented SARS-CoV-2 infection [8]. Aplastic anemia and resulting pancytopenia have been reported after some viral infections, including parvovirus B19 [18], EBV [19], CMV, varicella-zoster virus (VZV), human herpesvirus 6 (HHV-6), HIV, HAV, and HCV, and dengue [15]. By excluding all primary and secondary causes of AA, the diagnosis of BM-induced aplasia following SARS-CoV-2 infection was established in our case.

### Cross-reactivity of SARS-CoV-2 infection with fourth-generation p24 HIV antigen/antibody assay

Another enigma, in this case, is the cross-reactivity of SARS-CoV-2 infection with fourth-generation p24 HIV antigen/antibody assay. Previous researchers discovered a similarity between HIV and SARS-CoV-2 viral proteins by sequencing, which could explain the rare occurrence of false positivity of HIV chemiluminescent assays by chemiluminescent tests [20–22]. False positivity of fourth-generation p24 HIV antigen/antibody also has been reported during EBV infection [20]. Reactive HIV testing results may cause diagnostic dilemmas and have psychosocial concerns, including marital disharmony, rejection by family members, stigma, depression, and suicidal ideation, especially in developing countries. On the other hand, COVID-19 infection can mimic some clinical manifestations of HIV infection like prolonged fever, stomatitis, persistent oropharyngeal candidiasis, weight loss, and lymphopenia. Our country is classified as having a low prevalence of HIV [23], and therefore three consecutive positive tests are required to prove infection. According to the updated recommendations of the Iranian national guideline on managing HIV/AIDS in children [9], for the diagnosis of HIV infection, third or fourth-generation screening assays should be used for initial screening. The second and third tests must be taken on those with a positive screening test. If the second test is negative, the fourth-generation assay must repeat the tests on the same specimen. Nucleic acid testing should be considered on any specimens with indeterminate results on initial testing [9, 24]. To conclude, immunity against SARS-CoV-2 following natural infection might produce false-positive results with the fourth-generation HIV antigen/antibody screening test. To prevent misdiagnosis, physicians should be aware of this possibility and proceed to further laboratory analysis before making a definite diagnosis of HIV infection.

### Prognose and clinical outcome

Based on available case reports, SARS-CoV-2 infection may lead to AA relapse or a severe decrease in blood indices requiring prompt management [8]. Besides, regardless of disease phenotype (even in asymptomatic cases), SARS-CoV-2 infection could be associated with severe hematological abnormalities that may require hospitalization and a potential increase in mortality [6]. SARS-CoV-2-induced BM aplasia may be associated with secondary bacterial and fungal infections, as reported in our case.

## Data Availability

All data generated or analyzed during this study are included in this published article.

## Abbreviations

BM: Bone marrow
MDS: Myelodysplastic syndrome
MPN: Myeloproliferative neoplasm
NGS: Next-Generation Sequencing
DIC: Disseminated intravascular coagulation
PICU: Pediatric intensive care unit
RT-PCR: Real-time polymerase chain reaction
GM: Serum galactomannan tests
